# The impact of aging, sex, and exercise on brain mitochondria

**DOI:** 10.3389/fragi.2026.1920914

**Published:** 2026-10-12

**Authors:** Jianhua Zhang, Victor Darley-Usmar, Yun Li

**Affiliations:** 1 Department of Pathology, University of Alabama at Birmingham, Birmingham, AL, United States; 2 Department of Zoology and Physiology, University of Wyoming, Laramie, WY, United States

**Keywords:** age, brain, exercise, mitochondria, sex

## Abstract

Mitochondrial function is essential for brain health. Its age-related decline impairs neuronal resilience and increases risk for the development of neurodegenerative diseases. As sex is an important biological variable in both physiological and pathological brain function, in this mini review, we highlight emerging evidence of sex differences in mitochondrial content and function in the brain and what is currently understood about how they change with age. Furthermore, as physical exercise is considered a key mitigating factor in brain injury and the development of neurodegenerative diseases, we review studies that address the effects of exercise on mitochondrial function in the brain, with particular attention to those that include data from both sexes. The current understanding of the effects of sex differences and exercise on brain mitochondrial function over the lifespan is limited, but nonetheless some interesting insights are emerging. We discuss the key knowledge gaps and offer some suggestions for further research. These include defining the extent and specificity of mitochondrial alterations with age in different species, brain regions and cell types, and their modification by exercise, which is both important for advancing aging biology and for designing intervention strategies to promote healthy aging, to mitigate age-dependent cognitive decline and treat neurodegenerative diseases.

## Introduction

Mitochondrial function is essential for maintaining neuronal activity, metabolic homeostasis, and cellular stress responses in the brain (Picard and McEwen, 2014). Neurons are energy-dependent cells, requiring continuous ATP production to sustain synaptic transmission, ion gradients, and axonal transport (Engl and Attwell, 2015). For example, synaptic vesicle recycling and neurotransmitter release are tightly coupled to mitochondrial ATP generation, while calcium buffering during neuronal firing depends on mitochondrial capacity to regulate intracellular calcium levels (Billups and Forsythe, 2002; MacAskill et al., 2010; Vos et al., 2010). Disruptions in these processes can impair neuronal signaling and contribute to synaptic dysfunction, an early feature of cognitive decline and neurodegenerative disease (Sayehmiri et al., 2024).

With aging, mitochondrial function declines, accompanied by measurable alterations in genomic stability, bioenergetic capacity, and structural integrity (Lopez-Otin et al., 2013; Lopez-Otin et al., 2023). For example, studies in human skeletal muscle have reported an age-associated accumulation of mitochondrial DNA mutations and deletions, leading to impaired electron transport chain activity and decreased ATP generation (Short et al., 2005). In addition, increased production of reactive oxygen species and compromised antioxidant defenses contribute to oxidative damage to mitochondrial proteins, lipids, and DNA (Shigenaga et al., 1994). The repair of these deleterious modifications appears to decline with age due to deficiencies in mitochondrial quality control (mitophagy) and general autophagy (Sun et al., 2016). Functionally, these changes manifest as reduced respiratory capacity, altered substrate utilization, and impaired mitochondrial dynamics, all of which have been linked to age-related cognitive decline and neurodegenerative diseases including Alzheimer’s disease (Wang et al., 2020; Swerdlow, 2023; Stillman et al., 2020; Li et al., 2024; Singh et al., 2025).

Lifestyle changes can promote healthy aging (Cartee et al., 2016; Thomas et al., 2023). It has been shown that physical activity is strongly associated with preservation of bioenergetic capacity not only in the muscle but also in the brain (Burtscher et al., 2021). Importantly, exercise improves brain mitochondrial function, specifically ATP production in an insulin-dependent manner (Ruegsegger et al., 2019). In the context of neurodegeneration, exercise has also been reported to improve learning and memory, increase synaptic plasticity, and reduce neuroinflammation (Svensson et al., 2015). At the mechanistic level, exercise increases expression of key regulators of mitochondrial biogenesis in the muscle (Wright et al., 2007). In both brain and muscle it has been shown that exercise elevates mitophagy (Zhao et al., 2020; Liu et al., 2021). These results imply the existence of integrated responses across the whole body, and the maintenance of mitochondrial quality may be through the coordinated processes of mitophagy and mitochondrial biogenesis. Furthermore, exercise-induced systemic factors, including circulating proteins and metabolites, have also been shown to confer neuroprotection (Burtscher et al., 2021; Samadian et al., 2025; Lee et al., 2021). These observations suggest that peripheral adaptations contribute to mitochondrial remodeling in the brain.

Despite these advances, an important and relatively underexplored dimension of mitochondrial biology is the role of biological sex, as there have been relatively few studies in which sex is included as a specific biological variable. Sex differences in mitochondrial function have been observed in multiple tissues, including the brain, even under baseline conditions (Sarver et al., 2024). However, the experimental paradigms needed to define sex-dependent differences in the brain must also take into account how metabolic function changes depending on the anatomical brain region. This coupled with the expected differences in results related to the experimental design and methodology, makes designing and interpreting data in this area of research challenging. Nevertheless, given the significant health impact, it is important that the research community focuses on designing experimental paradigms that can define sex as a biological variable in determining how brain function changes with age. Emerging evidence suggests that males and females exhibit distinct trajectories for mitochondrial decline (Sarver et al., 2024; Adekunbi et al., 2025). In addition, it remains unclear how sex influences mitochondrial adaptive responses to interventions such as exercise, particularly in the aging brain. This represents a critical knowledge gap, as exercise is one of the most accessible and widely recommended strategies for promoting brain health (Stillman et al., 2020; Li et al., 2024; Singh et al., 2025).

In this short mini-review, we highlight current evidence supporting sex-specific differences in mitochondrial function, with a particular focus on how aging differentially affects males and females. We also discuss the limited number of recent studies suggesting that exercise-induced, or exercise-capacity-related mitochondrial adaptations vary by sex. Finally, we review some of the key knowledge gaps that could be prioritized in future research. A clearer understanding of these interactions will be essential for developing more targeted strategies to preserve mitochondrial function and promote healthy brain aging. We have assessed a broad range of literature from different fields and to aid the reader we summarize selected key articles in Tables 1 and 2 with a brief outline of methodology and key findings. The literature cited is constrained by the mini-review format and is not intended to be comprehensive but rather to exemplify the scope and key findings relevant to the area which have been performed to date.

**TABLE 1:** Age and sex effects on mitochondrial content and activity.

Species	Age	Sex	Tissue	Methods	Age and sex effect	Ref
Wistar rats	28, 60, 92 weeks old n = 20 each group	Male	Brain	Isolated mitochondria, biochemical methods	Unchanged mitochondria mass by age; ↓ activities in SOD2 activities to 62%, complex I-III by ∼30%, and complex IV by 28% by age; ↑ mitochondrial swelling by age	(Navarro and Boveris, 2004)
Wistar rats	6, 12, 18, and 24 mo old, n = 6–8 each group	Male and female, tested sex-by-age interaction	Brain	Isolated mitochondria, biochemical methods	↑ mtDNA (+237% females and +147% males from 6 to 24 mo), citrate synthase (CS) (+90% females, +48% males) and ATPase activity (+100% females and +66% males) by age; ↑ mtDNA in young males compared to females (50%); ↓ mitochondrial protein in mid-age and aged males compared to females (18%); ↓ CS activity in mid-age males compared to females (20%); ↓ ETC complex IV activity in males compared to females across age (∼20–70%)	(Guevara et al., 2011)
C57BL6 mice	6 weeks old, n = 6–8	Male and female	Brain	Isolated mitochondria using Oroboros Oxygraph-2K	↓ mitochondrial state 3 complex I and II in males compared to females (33%)	(Khalifa et al., 2017)
C57BL6 mice	2 mo old, n = 12 each sex	Male and female	Cortex	Seahorse extracellular flux bioanalyzer	No sex difference in, ETC complex I-IV	(Huynh et al., 2022)
C57BL6 mice	2.5 and 20 mo old, n = 10 per age and sex	Male and female, tested sex-by-age interaction	Cortex, hippocampus, cerebellum, and hypothalamus	Seahorse extracellular flux bioanalyzer	↓ activities in, ETC complex C-I, II and IV in both the cortex (∼40%) and hippocampus (30%–40%) in male by age; ↓ activities in, ETC complex C-I, II and IV in cortex (25%–35%) but not hippocampus in female by age; ↑ ETC complex C-II and C-IV activity in the hippocampus of young males (25%); No sex difference in the cortex, ETC complex of young animals; No sex difference in the cortex and hippocampal, ETC complex of old animals	(Sarver et al., 2024)
C57BL6 and SAMP8 mice	3 and 20 months and 2 and 10 months n ≥ 5 per sex and age	Male and female, Tested sex-by- age interactions tested	Hippocampus	Enriched mitochondrial fraction for mPTP, lysates for other assays	↑ 4-HNE immunostaining and MitoSox signal in aged male ↑ GSR protein in aged female ↓ATP content in aged female mitochondria ↓mitochondrial membrane potential in both sexes ↓complex I, III and IV protein levels by age in both sexes, complex II in male by age ↓Lonp1 proteolytic activity by age in both sexes	(Cicali et al., 2025)
Baboon	6.6–22.8 years, ∼26–91 years in human, total of 60	Male and female, tested sex-by-age interaction	Cortex	Seahorse extracellular flux bioanalyzer	↓ ETC activities in the cortex in male or combined sexes (not female alone) by age (10%–50% oldest compared to youngest); ↑ ETC I-IV activity in males compared to females across ages (10%–40%)	(Adekunbi et al., 2025)
Human	Age/Sex of 24/M, 67M, 73M, 77F, 85F, 86F, 94F	sex differences not explored	Cerebellum, temporal, frontal, occipital, parietal cortex and putamen	Dilution PCR	4977 nucleotide pair mtDNA deletion was ↑ in subjects over 80 compared to 67–77 in the cortex and putamen	(Corral-Debrinski et al., 1992)
Human	12 subjects age 26–85	sex differences not explored	Frontal cortex, superior temporal cortex, putamen and cerebellum	Enzymatic assays in crude mitochondrial extracts	Complex IV but not II activities ↓ with age, and the ↓ was not significant if normalized with citrate synthase activities	(Ojaimi et al., 1999)
Human	34 subject 0–87 years (median 50)	sex differences not explored	Hippocampal pyramidal cells and choroid plexus epithelial cells	SDH and COX enzymatic histology	There is an ↑ of COX deficient cells in these two cell populations with age	(Cottrell et al., 2001)

**TABLE 2:** Mitochondrial content and activity studies include exercise in aging, disease, injury and intrinsic aerobic capacity models, with potential inclusion of both sexes.

Species	Disease or injury model	Age	Inclusion of sexes	Exercise intervention	Duration	Tissue	Effect	Ref.
C57BL6 mice	​	8 weeks, n = 9–11	Male	Treadmill running	8 weeks	Cortex and hippocampus	↑ PGC1α and citrate synthase (CS) mRNA levels in several brain regions (15%–150%); ↑ mtDNA (40%–450%)	(Steiner et al., 2011)
C57BL6 mice	​	19 mo, n = 12	Male	Treadmill running	8 weeks	Cortex and hippocampus	↑ PGC1α protein levels (50%); ↑ mitochondrial DNA (30%); ↑ citrate synthase mRNA (20%)	(E et al., 2014)
Sprague-Dawley Rat	​	21 days, n = 6	Male	Treadmill running or Free-wheel running	12 weeks	Cortex	↑ aconitase and SOD2 activities (20%–50%); ↑ state 3 respiration (20%–45%), ↑ complex I, IV and complex V, PGC1α, Mfn1/2 protein levels (15%–50%)	(Marques-Aleixo et al., 2015)
C57BL6 mice	​	4 weeks and 24 mo, n = 7–8	Male	Treadmill running	3 weeks	Isolated mitochondria from the brain without cortex and striatum	↑ glutamate/malate linked state 3 respiration (25%), and CI-III activities (30%) in the old mice	(Gusdon et al., 2017)
C57BL6 mice	​	15 and 28 mo, n = 10	Male	Treadmill running	8 weeks	hippocampus	↑ glutamate/malate-, ADP- and succinate-supported respiration in both age groups (20%–40%)	(Park et al., 2025)
Lewis rat	​	6 months sedentary n = 10, 15 months sedentary n = 12, 15 months physical activity (PA) n = 12	Female	Access to a running wheel, allowed to run voluntarily for 9 months	9 months	Cortex and cerebellum	No difference in citrate synthase activity, protein levels of CI, II, III, IV, or V, no differences for complex I + III or complex II activity in the cortex ↑Complex IV activity in PA group in the cortex but not cerebellum No differences in PGC1α, NRF1, or TFAM, PINK1, Parkin, Mfn2, Opa1, Drp1, Fis1, SOD1, CAT, GPX1 ↑SOD2 in 15 months sedentary compared to 6 months in the cortex	(Mesquita et al., 2022)
Fisher 344 rats	​	8 weeks old	8 male and 8 female, analyzed sex × exercise interaction	Sedentary or treadmill	1 week acclimatization, 10 weeks treadmill training	hippocampus	↑ Complex II SDHA protein in female and ↓ Complex II SDHA protein in male exercise group, but no change in complex I, III or IV protein levels by exercise or sex	(White et al., 2025)
APP/PS1 mice	This is a disease model	3 mo, n = 9	Male	Treadmill running	12 weeks	Hippocampus	↓damaged mitochondria in CA1 region (∼50% by EM assessment) Restored APP/PS1 induced ↓ of PGC1α protein (50%) levels to that of WT levels	(Zhao et al., 2020)
Swiss-Webster mice	This is an injury model	4–6 weeks, n = 4–5	Male and female, analyzed injury x sex × exercise interaction	Low, moderate and high intensity exercise	72 h after controlled cortical impact injury (CCI)	Cortex	Low intensity exercise ↑ ATP linked oxygen consumption rate (OCR) only in females (40%),	(White et al., 2023)
Wistar rats with or without hypoxia-ischemia (HI) injury	This is an injury model	24 h after HI at postnatal day 7, n = 5–8	Pups of both sexes, analyzed injury x sex × treatment interaction	maternal exercise (daily swimming)	During gestation	Brain hemisphere	↑ mitochondrial, ETC C-II enzymatic activities in cortex and hippocampus from pups of both sexes (30%–50%, driven by without HI)	(Sanches et al., 2021)
Heterogeneous rats (N:NIH stock) bred into low-capacity runner (LCR) and high-capacity runners (HCR)	This is an intrinsic capacity model	18 and 24 mo, n = 4–8	Male and female, analyzed aging × strain interaction by 2-way ANOVA within sex.	LCR and HCR capacity	No further exercise	Half of the brain	↓ state 3 respiration in both sexes in 24 mo old compared to 18 mo old; HCR females exhibited ↑state 3 respiration compared to LCR females, especially in 18 mo (125%); HCR males exhibited ↓ state 3 respiration at 18 mo compared to LCR males (50%); ↑ C-II respiration in 24 mo females compared to 18 mo (25%), ↓ C-II respiration in HCR females compared to LCR females (50%); ↑C-II in HCR males compared to LCR at 24 mo (200%)	(Kugler et al., 2024)

## Sex differences in the age-dependent decline in brain mitochondrial function

Differences in mitochondria between sexes and across the lifespan have been widely investigated, although the outcomes show considerable variation (Sarver et al., 2024; Adekunbi et al., 2025; Navarro and Boveris, 2004; Guevara et al., 2011; Khalifa et al., 2017; Benavides et al., 2022; Acin-Perez et al., 2020; Huynh et al., 2022; Corral-Debrinski et al., 1992; Ojaimi et al., 1999; Cottrell et al., 2001). These differences are not unexpected given the range of species (rats, mice, primates, or humans) (Sarver et al., 2024; Adekunbi et al., 2025; Navarro and Boveris, 2004; Guevara et al., 2011; Khalifa et al., 2017; Benavides et al., 2022; Acin-Perez et al., 2020; Huynh et al., 2022; Corral-Debrinski et al., 1992; Ojaimi et al., 1999; Cottrell et al., 2001), sample preparation (mitochondrial isolation (Navarro and Boveris, 2004; Guevara et al., 2011; Khalifa et al., 2017) or use of whole cell extract from frozen tissues (Sarver et al., 2024; Adekunbi et al., 2025; Benavides et al., 2022; Acin-Perez et al., 2020; Huynh et al., 2022)), and the different techniques used to assess mitochondrial function (Sarver et al., 2024; Adekunbi et al., 2025; Navarro and Boveris, 2004; Guevara et al., 2011; Khalifa et al., 2017; Benavides et al., 2022; Acin-Perez et al., 2020; Huynh et al., 2022; Corral-Debrinski et al., 1992; Ojaimi et al., 1999; Cottrell et al., 2001). We outline here some emerging findings with a more detailed description of some key articles.

In a study using only male Wistar rats (28, 60 and 92 weeks of age) the effect of aging on isolated brain mitochondria was investigated. Key findings were that mitochondrial mass (protein/organ in mg/g) was unchanged, while the activities of Mn-superoxide dismutase, ETC complex I (C-I), and complex IV (C-IV), decreased with age. There was also increased mitochondrial size, fragility, and oxidative stress markers (Navarro and Boveris, 2004). These findings suggest that functional decline can occur independently of changes in mitochondrial abundance which in turn may be related to the declining efficiency of mitochondrial quality control with aging (Table 1, first row).

In a later study including both sexes, animals aged at 6–24 months were compared. As expected it was reported that, males had both a higher body and brain weight (Guevara et al., 2011). Consistent with the assumption that higher body or brain mass requires more energetic support (Kleiber, 1947), mitochondrial DNA levels in the brain also were higher in young males compared to females (6 months of age) (Guevara et al., 2011). Interestingly when normalized for the different basal weight between males and females it was evident that males had a lower brain/body weight ratio across the 6–24 months age span, and lower mitochondrial protein levels compared to females at both mid-age (12 months) and older age (24 months) (Guevara et al., 2011). In support of this data, suggesting a proportionally higher mitochondrial content in females at 12 months, citrate synthase activity, which is often considered a surrogate for mitochondrial content, was higher in females compared to males (Guevara et al., 2011). Furthermore, ETC complex IV activity was reported to be higher in females than males in all the ages tested (Guevara et al., 2011) and Complex V activity was higher in the females at 12 and 24 months of age (Guevara et al., 2011). In this study age × sex interaction analyses were performed and found significant for body weight, brain/body weight ratio, brain total protein, brain total DNA, brain total protein/total DNA, and citrate synthase activity (Guevara et al., 2011). These data strongly highlight the fact that there are fundamental differences in how bioenergetic function shows sex-dependent differences in the brain.

In the same study, aging was found to be associated with decreased brain/body weight ratio in both sexes (Guevara et al., 2011). Interestingly, and perhaps unexpectedly, mitochondrial DNA, citrate synthase activities, and complex V activities were increased with age in the brain (Guevara et al., 2011), suggesting that mitochondrial content and most likely bioenergetics show age-dependent adaptations to changing metabolic needs. It is important to note that at this stage we do not know whether the apparent increase in mitochondrial energetics reflects and healthier mitochondria. For example, this change in bioenergetic markers may be due to a compensatory response to declined mitochondrial efficiency, or could arise from a decreased mitochondrial turnover (Guevara et al., 2011). In addition, it was reported that not all mitochondrial, ETC enzymes exhibited increased activity. For example, complex IV activity remained unchanged across the age range (Guevara et al., 2011). There was an increase of state 3 and state 4 oxygen consumption but not Respiratory Control Ratio (RCR) with age with isolated mitochondrial preparations and pyruvate/malate as substrates, while there were no sex differences observed (Guevara et al., 2011). Complex IV protein subunits seem to be differentially regulated by age and sex, as COXIV protein levels were not changed by age or sex, while COXII was increased with age and the increase was higher in females (significant age × sex interaction) (Guevara et al., 2011) (Table 1 row 2). The reported increase of mitochondrial complex V and citrate synthase activities, as well as selective, ETC protein subunit levels, with age is perhaps counter-intuitive as mitochondrial deficits are generally considered a hallmark of aging (Lopez-Otin et al., 2013). However, changes in mitochondrial fuel selection and the impact on inflammatory signaling will not be evident from this experimental paradigm using isolated mitochondria with supplied substrates (Wang et al., 2026; Palsson-McDermott and O'Neill, 2025; Marchi et al., 2023; Hill et al., 2012; Hill et al., 2019). Clearly, measurement of a single parameter such as mitochondrial DNA, protein, citrate synthase activity, or even complex V activity levels is not a surrogate for all mitochondrial functionality. A more integrated approach using metabolomics and proteomics will be beneficial in future studies.

Studies using mouse brains supported that sex-dependent differences in mitochondrial function may vary depending on the brain regions being analyzed (Sarver et al., 2024; Khalifa et al., 2017). In young C57BL6 mice (6 weeks of age), it was reported that mitochondria isolated from female mice had higher state 3 complex I and complex II dependent respiration than males (Khalifa et al., 2017). In support of the previous study, quantification using electron microscopy showed more mitochondria in the female brain compared to males, while mitochondrial morphology was similar between sexes (Khalifa et al., 2017) (Table 1 row 3). In comparison, a later study using cortical extracts that had not undergone mitochondrial isolation, reported no apparent sex differences in, ETC activities in 2 month old young mice (C57BL/6) (Khalifa et al., 2017; Huynh et al., 2022) (Table 1 row 4). These apparent discrepancies have been at least partially resolved by studies which examine specific brain regions as discussed next.

Regional specificity in mitochondrial function is supported by findings that hippocampal—but not cortical—mitochondrial activity differs between sexes in young mice (Sarver et al., 2024). In multi-tissue analyses, aging was found to reduce mitochondrial activity across brain regions, although the magnitude and pattern differed between sexes (Sarver et al., 2024). Using a systematic comparison of young vs. old, male vs. female mitochondrial, ETC I, II and IV activities in young (2.5 months old) and old (20 months old) mice of both sexes were measured (Sarver et al., 2024). In the 4 regions of the brain assessed (cortex, hippocampus, cerebellum, and hypothalamus), only the hippocampus exhibited sex differences in, ETC C-II and C-IV with higher C-II and IV in males compared to females in the young mice (Sarver et al., 2024), while cortex, ETC complexes were not different between sexes (Sarver et al., 2024), replicating previous findings (Huynh et al., 2022). In the older mice, ETC C-I, II and IV activities in the cortex and hippocampus were similar between sexes. While, ETC C-I, II and IV activities were all lower in older compared to younger male mice in the cortex and hippocampus, they were lower in older compared to younger female mice only in the cortex but not in the hippocampus (Sarver et al., 2024). This study highlights how brain region and sex differences can impact the measurement of mitochondrial, ETC activities with age and again emphasizes how mitochondrial function can exhibit sex and brain-region dependent differences (Table 1 row 5).

Sex differences in age-related mitochondrial changes have also been reported in mitochondrial membrane potential and mitochondrial protease activities. One study focused on the hippocampus using C57BL/6J mice and SAMP8 mice as an aging model (Cicali et al., 2025). It was reported that oxidative stress was higher in aged males, while mitochondrial permeability transition pore function was higher in aged females. Furthermore, there was a significant decrease in the mitochondrial protease Lonp1 activity (Cicali et al., 2025). These observations are intriguing and suggest that mitochondrial protein turnover may also be an important age-dependent factor which has received relatively little attention (Table 1 row 6).

Studies in the cortex in non-human primates across a wide age range further demonstrated an age effect on mitochondrial function (Adekunbi et al., 2025) (Table 1 row 7). The interaction term between age and sex on mitochondrial function was not significant, although the sample size (total of 60) may not be sufficiently powered for the interaction analysis (Adekunbi et al., 2025). Secondary analysis in which the data was stratified by sex revealed that the association between age and each of the, ETC activities differed between sex, and the overall age-related decline in mitochondrial, ETC activities was largely driven by males (Adekunbi et al., 2025). Further, sex-based analysis of mitochondrial activity independent of age showed that males had higher respiration compared to females (Adekunbi et al., 2025). In baboons, body weight was not a significant predictor of mitochondrial, ETC activities, while walking speed measured 2 years prior to necropsy was positively correlated to mitochondrial, ETC activities in the cortex (also largely driven by males) (Adekunbi et al., 2025). This is interesting, as epidemiological studies clearly demonstrated that muscle and cognitive frailty, including depression, co-manifest in aging human populations (Vaughan et al., 2015).

Studies on *postmortem* human brains with age stratification are limited for obvious reasons such that the post-mortem brain specimens which have been examined are in general from older individuals. Even in the young, most were samples also associated with trauma, severe injury or end stage diseases. Nevertheless, one interesting study with 7 *postmortem* human brains found that, using dilution PCR, the common 4977 nucleotide pair mtDNA deletion was higher in subjects over 80 years of age, compared to those aged 67–77 in the cortex and putamen (Corral-Debrinski et al., 1992). This study supports the idea that age leads to impaired mtDNA integrity (Table 1 row 8).

In a different study with 12 subjects aged 26–85 it was found that complex IV but not complex II enzymatic activities decrease with age in frontal cortex, superior temporal cortex, putamen and cerebellum (Ojaimi et al., 1999). The decline of complex IV activities was no longer significant if normalized to citrate synthase activities, consistent with an overall decrease of mitochondrial content but could also represent changing levels and activities of the TCA cycle (Ojaimi et al., 1999) (Table 1 row 9). In a histology study of 34 brains (0–87 years of age), it was shown that complex IV-deficient cells were increased in hippocampal pyramidal neurons and choroid plexus epithelial cells (Cottrell et al., 2001) (Table 1 row 10). These studies begin to address whether there is an age-dependent decline of mitochondrial, ETC activities in human brains.

Overall, the observed sex differences and age-related declines in mitochondrial function are likely influenced by sampling of specific brain regions, and the methodologies used to prepare and measure mitochondrial function. Furthermore, the mechanisms of potential sex and age effects on brain mitochondrial function can be multi-faceted. For example, the brain not only receives sex hormones from the peripheral tissues through the blood-brain barrier, but also synthesizes sex hormones locally (Torrens-Mas et al., 2020). With aging the levels of sex hormones, including estrogen, progesterone, testosterone, and related metabolites in the brain, decrease (Torrens-Mas et al., 2020; Caruso et al., 2013; Rosario et al., 2009). Given that estrogens and androgens, through their nuclear receptors, can regulate the expression of PGC1α, uncoupling proteins, citrate synthase, cytochrome c and complex IV subunits (Torrens-Mas et al., 2020; Ahmad and Newell-Fugate, 2022; Liu et al., 2019; Yin et al., 2023), it is likely that sex hormones can impact mitochondrial function in the brain. These mechanisms are complex since they are also influenced by genetics, lifestyle and both central nervous system and peripheral tissue structure, function and fitness. Studies investigating how estrous status, reproductive senescence, ovariectomy, menopausal transition, and age-related changes in androgen concentrations will be informative in this regard. With aging multiple factors may contribute to mitochondrial adaptation and maladaptation, including but not limited to genomic instability, chronic inflammation, autophagy deficits, altered intercellular communication, cellular senescence and stem cell exhaustion (Lopez-Otin et al., 2013; Lopez-Otin et al., 2023; Barbosa et al., 2018).

## Sex differences in exercise-induced mitochondrial adaptation in aging brains

Exercise is a powerful modulator of aging, with beneficial effects on cognition, neuroplasticity, and neuroprotection (Cartee et al., 2016; Zhao et al., 2020; Lee et al., 2021; Ruegsegger and Booth, 2018; Kramer et al., 2006; Churchill et al., 2002; Grill and Gillen, 2025; Ornish et al., 2024; Baker et al., 2025; Wang et al., 2022; Jian et al., 2022; Zhang et al., 2022; Mehla et al., 2022; Zhao et al., 2024; Zeng et al., 2020; Zhu et al., 2022; Koga et al., 2014; Gratuze et al., 2017; Perez Garcia et al., 2023; Cai et al., 2025). However, relatively few studies have directly examined how exercise influences mitochondrial function in the aging brain, and even fewer have addressed sex-dependent responses (Table 2 and the references therein). We highlight some key studies below.

In an effort to investigate the effects of exercise on brain mitochondria, young male mice were subjected to treadmill running until fatigue. This exercise was associated with an increase in markers of mitochondrial biogenesis in the cortex and hippocampus, including PGC1α and citrate synthase mRNA levels, and mitochondrial DNA content (Steiner et al., 2011) (Table 2 row 1). A similar exercise regimen as above reported that citrate synthase mRNA (sampled from the left hippocampus), and PGC1α protein and mitochondrial DNA (samples from the left hemisphere cortex) were elevated by exercise in the old mice again supporting a role for mitochondrial biogenesis in response to aging and exercise (E et al., 2014) (Table 2 row 2).

The studies discussed have also been complemented by reports that show that brain mitochondrial function is enhanced by exercise (Marques-Aleixo et al., 2015; Gusdon et al., 2017; Park et al., 2025). As such, most studies have focused exclusively on males, limiting conclusions about sex-specific adaptations. Studies in rats (Marques-Aleixo et al., 2015) and mice (Gusdon et al., 2017; Park et al., 2025) both found enhanced brain mitochondrial respiration after treadmill running or free-wheel running exercise, while the specific changes in, ETC complex activities were different in these studies, likely due to differences in the exercise regimen, species, age of the animals, brain regions and measurement methodology (Table 2 row 3–5).

In a series of experiments involving only female animals, the impact of voluntary wheel running physical activities for 9 months on brain mitochondrial function in female rats was investigated. There were no age or exercise-dependent effects on citrate synthase activity, protein levels of, ETC complexes CI, II, III, IV, or V, levels of mitochondrial biogenesis transcription factors PGC1α, NRF1, or TFAM, levels of fission and fusion proteins Mfn2, Opa1, Drp1, or Fis1, levels of mitophagy proteins PINK1 or Parkin, levels of antioxidants SOD1, CAT, GPX1, activities of complex I + III or complex II in the cortex or cerebellum. There was an increase in complex IV activity in the physical activity group, and there was an increase of SOD2 protein at 15 months of age compared to 6 months of age in the cortex of sedentary group but not cerebellum (Table 2 row 6) (Mesquita et al., 2022). One conclusion might be that this protocol for voluntary wheel running was insufficient to induce robust mitochondrial changes. This may be the case since a 10-week treadmill running study, using both male and female Fisher 344 rats, showed an increase in complex II SDHA protein levels in females, but a decrease in complex II SDHA protein levels in the male exercise group. No differences were observed in complex I, III or IV protein levels between sexes or between sedentary and exercise groups (Table 2 row 7) (White et al., 2025). Exercise intensity may then play a role in sex-dependent responses to exercise.

In addition to the studies of effects of exercise in wild-type animals, there have been some investigations using disease and injury models. In a model of Alzheimer’s disease, male APP/PS1 mice (a model with amyloid pathology) were subjected to treadmill exercise (Zhao et al., 2020). It was found that exercise mitigated the decrease in PGC1α associated with APP/PS1 expression, consistent with a positive effect of exercise on brain mitochondrial biogenesis, and a potential mechanism for exercise in neuroprotection (Zhao et al., 2020) (Table 2 row 8).

An interesting and topical question is whether exercise can improve recovery from brain trauma and whether this effect is sex-dependent. In a study with male and female Swiss-Webster mice (4–6 weeks of age), low, moderate or high intensity exercise regimens were applied to mice 72 h after controlled cortical impact (CCI) injury or sham surgery. Mitochondrial function was assessed with cortical biopsy punches. Low intensity exercise improved basal and ATP linked oxygen consumption rate only in females, while males exhibited decreased basal and ATP linked oxygen consumption rates after high intensity exercise. This study demonstrated how cortical mitochondrial adaptation to exercise after CCI appears to be different between sexes (White et al., 2023). In a study of neonatal hypoxia-ischemia injury, Wistar rats subjected to maternal exercise (daily swimming) resulted in pups of both sexes exhibiting higher rat brain hemisphere mitochondrial, ETC C-II enzymatic activities after neonatal hypoxia-ischemia injury (Sanches et al., 2021) (Table 2 row 9–10).

The association of aerobic exercise capacity and brain mitochondrial activities was investigated in low-capacity runners (LCR) and high-capacity runners (HCR) which were selected by breeding genetically heterogeneous rats (N:NIH stock) (Koch and Britton, 2001). Using 18 and 24-month-old male and female LCR and HCR rats, mitochondrial function was measured in brain tissues. It was found that state 3 and state 4 respiration was lower in 24 months old compared to 18 months old rats in both sexes, independent of the runner capacities. HCR females exhibited higher state 3 and state 4 respiration compared to LCR females at both ages, while HCR males exhibited lower state 3 respiration at 18 months of age compared to LCR males. While 24 months females exhibited higher C-II respiration compared to 18 months, HCR females exhibited lower C-II respiration compared to LCR females. In males HCR was associated with higher C-II compared to LCR at 24 months of age (Table 2 row 11). This exploratory study again provides indirect evidence of sex differences in mitochondrial function related to intrinsic aerobic capacity (Kugler et al., 2024).

Mechanistically, exercise-induced improvements likely involve multiple pathways, including enhanced mitochondrial biogenesis, improved mitochondrial quality control, and systemic signaling between peripheral tissues and the brain (Zhao et al., 2020; Steiner et al., 2011; E et al., 2014; Marques-Aleixo et al., 2015; Gusdon et al., 2017; Rocchi et al., 2017; He et al., 2012). Sex-specific influences may arise from differences in hormonal regulation and metabolic flexibility (Torrens-Mas et al., 2020; Caruso et al., 2013; Rosario et al., 2009; Ahmad and Newell-Fugate, 2022; Liu et al., 2019; Yin et al., 2023). Overall, while exercise supports mitochondrial function in the brain, how mitochondrial adaptation to exercise in the brain across lifespan is affected by sex still needs further investigation.

## Future directions and conclusions

Despite increasing interest and a growing number of studies on sex, aging, and brain mitochondria (Tables 1 and 2), major gaps remain in understanding how mitochondrial function declines with age, in different cell types or brain regions, how mitochondrial changes differ between sexes, and how exercise modifies mitochondrial function. Addressing these questions will require more integrative and mechanistic approaches. These points are elaborated below:

1. One key gap is insufficient integration of sex as a biological variable. Although sex differences are increasingly recognized, few studies have sufficient statistical power to investigate sex differences in exercise-induced mitochondrial responses across the lifespan.
2. Current studies have mostly been limited in the number of region-specific analyses in organs such as the brain. For example, many studies rely on whole-brain or cortical measurements, which obscure region-specific differences. This is particularly important given the selective vulnerability of brain regions to the pathological effects of neurodegenerative diseases.
3. Our understanding of the impact of cellular heterogeneity on the interactions between sex-, age- and exercise-dependent regulation of mitochondrial function is in its infancy. Different cell types in the brain have distinct metabolic demands and mitochondrial properties, yet most studies use bulk tissue approaches so this information is lost. Advances in single-cell bioenergetic/metabolic methodologies will be critical to fill this gap.
4. There is also a need for greater mechanistic clarity regarding how exercise influences mitochondrial function. While exercise improves mitochondrial outcomes, the relative contributions of biogenesis, mitophagy, and mitochondrial dynamics remain unclear. Systemic factors also play an important role. Peripheral signals induced by exercise, including circulating factors, influence brain function and mitochondrial adaptation. From what little we do know it is clear that these pathways are likely to differ between sexes.
5. Finally, translating findings from animal models to humans remains a major challenge. While animal studies provide mechanistic insights, human studies are needed to establish relevance and inform clinical interventions. Effects of different exercise mode, intensity, frequency, and duration need to be further studied.

Addressing these gaps will require multidisciplinary approaches integrating molecular, physiological, and system-level analyses. A better understanding of sex-, region-, and cell-specific mitochondrial biology in aging and in response to exercise may enable more precise strategies to promote brain health during aging.
